# School Menu Review Programme (PReME): evaluation of compliance with dietary recommendations during the period 2006–2020 in Catalonia

**DOI:** 10.1186/s12889-022-14571-1

**Published:** 2022-11-25

**Authors:** Maria Blanquer-Genovart, Maria Manera-Bassols, Gemma Salvador-Castell, Oriol Cunillera-Puértolas, Conxa Castell-Abat, Carmen Cabezas-Peña

**Affiliations:** Public Health Agency of Catalonia, Subdirectorate General for Health Promotion, Ministry of Health, Government of Catalonia, Roc Boronat, 81-95, Barcelona, 08005 Spain

**Keywords:** School meals, School menus, School canteen, School cafeteria, Dietary recommendation compliance, Meal assessment, Meal planning, Child nutrition

## Abstract

**Background:**

The School Menu Review Programme (PReME) has been offering complimentary revisions of meal plans to all schools in Catalonia since 2006. This study aims to assess the evolution of compliance with PReME’s recommendations in the meals provided by school cafeterias in Catalonia during the period 2006–2020.

**Methods:**

Pre-post study with a sample of 6,387 meal plans from 2221 schools assessed during the period. The information was collected mainly by public health specialists within the annual technical and sanitary inspection of school kitchens and cafeterias. Meal plans were evaluated by Dietitian-Nutritionists team according to the criteria of the National Health System’s “Consensus document on nutrition in schools” and the Public Health Agency of Catalonia’s current guide “Healthy eating at school”. Reports were sent to each participating school. A few months later, a new meal plan and another questionnaire were collected and evaluated in comparison with the first meal plan. Compliance with the recommendations was analysed based on the type of canteen management and the school category.

**Results:**

Compliance improved during the study period. The percentage of schools that complied with dietary recommendations in relation to the five PReME indicators (fresh fruit, pulses, daily vegetables, fresh food and olive oil for dressing) has steadily increased since PReME began, (over 70% in all indictors; *p* =  < 0.001), with variations depending on school category and cafeteria management. Furthermore, an improvement in the levels of compliance with de recommended food frequencies was observed. with statistically significant differences for all items (*p* < 0.001), except for pulses whose compliance had been high since the beginning of the study (*p* = 0.216).

**Conclusions:**

The positive evolution in compliance with PReME’s recommendations provides evidence of the programme’s effectiveness, with an improvement in the quality of school meals delivered in Catalonia.

## Background

During the last two decades, the scientific evidence available on the importance of diet as a health determinant has grown considerably [1–12]. One third of children and adolescents have an unbalanced diet [13, 14]. This is considered the main risk factor for diet-related non-communicable diseases (NCDs) [15–18]. 

In recent years, due to social, economic and political changes [19] that have favoured the creation of obesogenic environments [16, 20–22], the prevalence of childhood obesity has reached epidemic figures both globally [23–28], and in Europe [29] and Spain [25, 30, 31]. Given its association with serious health consequences and its persistence into adulthood, this development is very concerning. In Catalonia, according to the *Health Survey of Catalonia* 2020, 24.2% of the population aged 6 to 12 was overweight and 11.7% obese [32].

Behaviours acquired in childhood persist over time and can influence future health and during the first thousand days of life, along with the school age period, the foundations of health are laid [33–39]. An integrated-school approach to healthy eating can provide children and adolescents with both the opportunity to learn food and nutrition skills as well as how best to implement them both within and outside the school setting [40]. As such, and taking into account the links between health and education [40], schools are a key setting for performing interventions aimed at reinforcing and modifying dietary habits, and health promotion efforts in this setting could have a broader impact on dietary behaviours as school meals can provide a foundation for educating children on nutrition, environmental responsibility and food safety [29, 41–54]. For the approach and prevention of food-related diseases WHO recommends multilevel interventions. In the school setting this could include reinforcing restrictions on accessibility to foods and beverages with low nutrient density and increasing access to foods with high nutrient density in spaces frequented by children [55, 56].

With the aim of reducing global morbidity and mortality associated with diet- and physical activity-related NCDs, the World Health Organization Global Strategy on Diet, Physical Activity and Health adopted by the World Health Assembly in 2004 calls for global, regional and local action to improve diet and increase physical activity. Among other proposals, it urges member state governments to adopt school policies and programmes to support healthy eating and physical activity. In alignment with these guidelines, Spain developed the Strategy for Nutrition, Physical Activity and Obesity Prevention (NAOS) in 2005. In Catalonia, in response to the increase observed in the prevalence of obesity, and in accordance with WHO’s global strategy and the NAOS strategy, the Comprehensive Plan for the promotion of health through physical activity and healthy eating (PAAS) was developed in 2006, led by the Ministry of Health’s Public Health Agency of Catalonia (ASPCAT) with the aim, among others, of raising public awareness of the problem of obesity and promoting initiatives that contribute to the adoption of healthy lifestyles, especially among children and young people.

In Spain, the first regulations for the operation of school cafeteria were drawn up in 1954. In 1992, the 24 October 1992 Order of the Ministry of Education and Science was approved, transferring powers in educational policies to the autonomous regions [57]. Royal Decree RD/160/1996 of 14 May stipulated that the cafeteria service in state-run schools is the responsibility of the Catalan Ministry of Education [58]. Subsequently, Law 17/2011 on food safety and nutrition established that the competent authorities would ensure that the meals served in nursery, primary and secondary schools would be varied, balanced and adapted to the nutritional needs of each age group [59].

In Catalonia, and within the framework of PAAS, an agreement was signed between the Ministries of Health and Education in 2006 to offer complimentary revisions of school meal plans, known as the School Menu Review Programme (PReME), with the aim of improving the quality of the meals offered in school cafeteria. PReME is structured in 3 phases:

- Phase 1. Initial evaluation by dietitian-nutritionists of a monthly meal plan and preparation of the report with suggestions for improvement.
- Phase 2. Follow-up of the actions taken by schools after receipt of the initial report. This phase began in 2012, and is offered to all schools that have completed phase 1.
- Phase 3. Sensory assessment by PReME staff of school meals and the cafeteria. This was launched on a pilot basis in 2015, and is carried out in schools that have completed Phases 1 and 2.

To date, there is no official legislation or punitive measures that make compliance with the PReME recommendations mandatory. Currently, the only incentive lies in the fact that the centers serving menus or the catering companies managing the school menus can obtain a satisfactory PReME report. This can be gratifying for the center’s management team, the associations of student families, as well as for the catering companies or local authorities. A satisfactory report can promote the continuity of the company or professionals who manage the menu service, while guaranteeing families that their children receive adequate and nutritious food.

Four Dietitian-Nutritionists (DNs) at ASPCAT’s central services and the public health specialists (PHSs) working in the regional teams that carry out the annual technical and sanitary inspection of school kitchens and cafeteria took part in the programme.

The aim of this study was to assess the evolution of compliance with PReME’s recommendations for meals provided by school cafeteria in Catalonia covering the programme’s implementation in 2006 until 2020. Possible differences in compliance to recommendations were analysed, based on who provided the service and the type of school category, as well as the availability of special menus, based on the type of school category.

## Methods

### Study design

Pre-post study designed to assess school meals in Catalonia.

In Catalonia, 2332 primary schools (most secondary schools do not offer cafeteria service) were able to join the PReME programme during the period 2006–2020. The sample is made up of data and assessments obtained from the evaluation of 6,387 school meal plans and their respective questionnaires in PReME phases 1 and 2, conducted between 2006 and 2020. Table 1, shows the main characteristics of the centers involved in the study.

**Table 1:** Description of the main characteristics of schools participating in the School Menu Review Programme (PReME), 2006–2020

	Phase 1
	*N* = 2221
	n (%)
School category	
*State*	1772 (79,8)
*Private or subsidised*	449 (20,2)
Educational level	
*Primary*	1536 (69,2)
*Primary or secondary*	685 (30,8)
Type of catering service	
*Own kitchen*	393 (17,7)
*Company in school*	1012 (45,6)
*Company with central kitchen*	815 (36,7)

For the present study, data of all schools that took part in the PReME during the period 2006–2020 were analyzed. Schools in the city of Barcelona were excluded, as they are managed by the Barcelona Public Health Agency. The programme was offered to all primary and secondary schools, whether state, subsidised or private, through the publication of the programme’s availability on the ASPCAT website and during the course of hygiene and sanitary inspections that were carried out. Since 2006, the PReME programme has been featured in a separate section of the ASPCAT website. As such, the requests for school meal reviews were received spontaneously. However, from 2009 onwards, the PReME programme was also offered to all schools visited by public health specialists throughout Catalonia during the course of hygiene and sanitary inspections conducted in school kitchens and cafeterias. An assessment was offered every three years in order to cover the maximum number of schools in Catalonia, which also allowed schools enough time to implement improvement strategies. Assessments could be more frequent if warranted by special circumstances (change of kitchen management, change of catering company, specific problems, etc.). A 4-week meal plan was collected for each seasonal cycle (spring–summer and autumn–winter), along with the information requested in an initial questionnaire.

### Sources of information

The information obtained in the PReME programme is coded in two phases.

In phase 1, the schools that spontaneously ask to take part in the programme and the PHSs that carry out the inspections in the schools send the application questionnaire and the meal plans (four weeks of the summer season and four weeks of the winter season) to the central services’ DN team. The data detailing the features of the school, school cafeterias, characteristics of the meal plan and the results of the meal assessments are entered in an Access database by the DN team. The DN team then generates a report with suggestions for improving meal planning, in accordance with the criteria of the National Health System’s “Consensus document on nutrition in schools (DoCACE)” (NAOS, 2010) [60] and the ASPCAT’s guide “Healthy eating at school” (with the version available at the time meals were assessed), which comprises the initial report. It is sent to all the stakeholders (school, parent-teacher association, catering company, and sometimes also the municipal council and the county council).

Phase 2, the follow-up phase, which began in 2012, is offered to all schools that have completed phase 1. Accordingly, the initial report is resent to the schools, together with the invitation to submit, within 2–3 months, a new 4-week meal plan for each season and a new questionnaire that will be evaluated. In the case of schools that take part in this second phase, the new meal plans are assessed and the DN team draws up a follow-up report, based on the same structure and criteria as the initial report.

### Study variables

For the present study, the application questionnaires provided the school’s identifying data (category – state, private or subsidised –, educational level – ‘primary’ or ‘primary and secondary’ –, contact details, etc.); information about the cafeteria service (number of students, number of students using the cafeterias, cafeteria management and type of catering service: a) Own kitchen- schools with their own kitchen that plan the meals, b) Company in school—external company that cooks at the school, c) Company with centralized kitchen—external company supplying schools from a centralized kitchen–); presence of food and beverage vending machines; types of oils used for cooking, frying and dressing; and menus for special situations such as illnesses (allergies or intolerances) or ethical or religious issues (pork-free, meat-free and vegetarian).

Based on the DN team’s evaluation of each school’s monthly meal plan, information was obtained about the weekly frequency of foods offered in the first courses: rice, pasta, pulses, salads and vegetables (including potatoes); in the second courses: meat, fish, eggs, plant-based protein; as side dishes: salad, potatoes and other side dishes (pulses, pasta, rice, cooked vegetables, mushrooms, etc.); and as desserts: fresh fruit, dairy and other desserts (canned fruit, nuts) along with data about fried foods, both in main courses and side dishes. Information was also collected on the number of precooked foods and sweet desserts served per month. The evaluation of compliance with the PReME recommendations was based on the description of the dishes, ingredients and culinary technique used in the meal plans. Table 2 shows the items evaluated and the recommended frequencies of foods that were applied in PReME during the study period. Compliance was considered to be adequate when the food count and cooking technique offered in the meal plan coincided with the recommendations of the current PReME guide and year: 2006 recommendations for meal plans received from 2006 until August 2010; 2010 recommendations for meal plans received from September 2010 until August 2015; 2015 recommendations for meal plans received from September 2015 until August 2016; 2016 recommendations for meal plans received from September 2016.

**Table 2:** Changes in the recommended food frequencies implemented in the School Menu Review Programme (PReME), 2006–2020

Foods and cooking techniques	Recommended portions per week (5 days)			
	2006	2010	2015	2016
***First course***				
Rice	1–2	1	1	1
Pasta	1–2	1	1	1
Pulses	1–2	1–2	1–2	1–2
Potato^a^	0–2	-	-	-
Salads and vegetables		1–2	1–2	1–2
Cooked	1–2			
Uncooked	0–1			
***Second course***				
Fish	1–2	1–3	1–3	1–3
Meat	2–3	1-3 (lean)/ 0–1 (fatty) –– In total, maximum 3 per week	1–3 (lean)/ 0–1 (fatty) –– In total, maximum 3 per week	1-3 (white)/ 0–1 (processed or red) –– In total, maximum 3 per week
Eggs	1–2	1–2	1–2	1–2
Plant-based protein	-	-	-	0–5
Precooked	-	0–3 per month	0–3 per month	0–3 per month
Fried	1–2	1–2	0–2	0–2
***Side dishes***				
Salad	3–4	3–4	3–4	3–4
Potatoes	0–2	1–2	1–2	1–2
Other	0–1			
Fried	1–2	0–1	0–1	0–1
***Dessert***				
Fresh fruit	3–4	4–5	4–5	4–5
Dairy products	1	0–1	0–1	0–1
Other	0–1			
Non-fresh fruit	-	-	0–1	0–1
Sweets	-	-	0–1 per month	0–1 per month

^a^ Since 2010, it has been recommended to always serve potatoes in combination with other vegetables

Five PReME indicators were established as key variables for evaluating the quality of the meal plans: fresh fruit for dessert, pulses as a first course, vegetables on the daily menu, fresh food (raw fruit and/or vegetables) in the daily menu, and olive oil for dressing (Table 3). These data were also assessed by the DN team based on the school’s meal plan. The data for fresh fruit for dessert and pulses were the percentage of weeks that the school complies with the recommendation in the meal plan provided. With respect to the daily presence of vegetables and fresh food in menus, unique variables were defined that integrated the information obtained from the evaluation of the monthly meal plan. In this case compliance was considered adequate (yes/no) when the meal plan showed fewer than five days per month without including either of these items. The data on the use of olive oil for dressings was extracted from the application questionnaires that had been completed by the school (yes/no).

**Table 3:** Description of the School Menu Review Programme (PReME) indicators and recommendation for compliance

PReME indicator	Recommendation
1. Fresh fruit for dessert	4–5 days with fresh fruit for dessert
2. Pulses as a first course	1–2 days with pulses as a first course
3. Vegetables in the daily menu	≤ 5 days without vegetables/month
4. Fresh food (fruit or vegetables) in the daily menu	≤ 5 days without fresh food/month
5. Olive oil for dressing	Confirmed use of olive oil for dressing

### Statistical analysis

The PReME indicators are described in terms of the absolute and relative frequency of schools that comply with the indicator in the case of monthly indicators (with regard to vegetables, fresh food and olive oil), and as means and standard deviations in the case of indicators based on the percentage of weeks complying with recommendations (for fresh fruit and pulses). The same statistics are provided for the other variables depending on whether they are binary/categorical or numerical, respectively.

In order to evaluate possible differences between years, or according to school category or type of kitchen, bivariate chi-squared tests were used for categorical variables, and t-test for numerical variables (ANOVA for the comparison between years).

A significance level of 0.05 was considered as an indicator of statistically significant differences. Data was compiled in an Access database and analysed with R statistical software, version R 3.6.1.

## Results

**Fig. 1:**
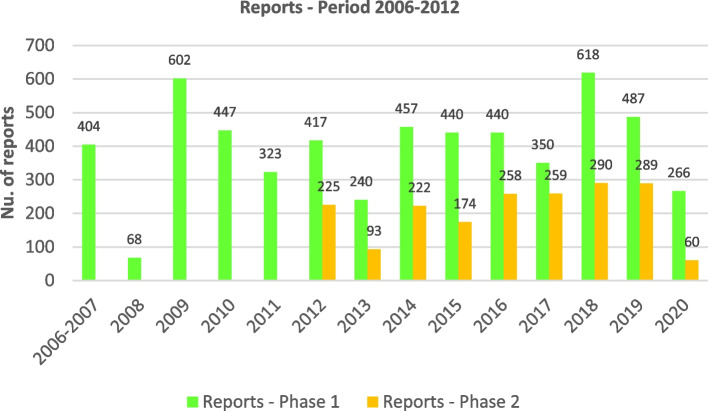
Evolution of the number of initial (phase 1) and follow-up (phase 2) review reports carried out in the School Menu Review Programme (PReME), 2006–2020

Of the 6,387 meal plans received (from 2221 schools) and assessed during the period 2006–2020, 4,742 initial reports were generated for phase 1 of the programme and 1,645 follow-up reports for phase 2 (phase 2 started in 2012) (Fig. 1). A total of 75.4% of the meal plans were from state schools and 88.9% are from primary schools; their cafeterias were used by a total of 788,971 pupils.

**Fig. 2:**
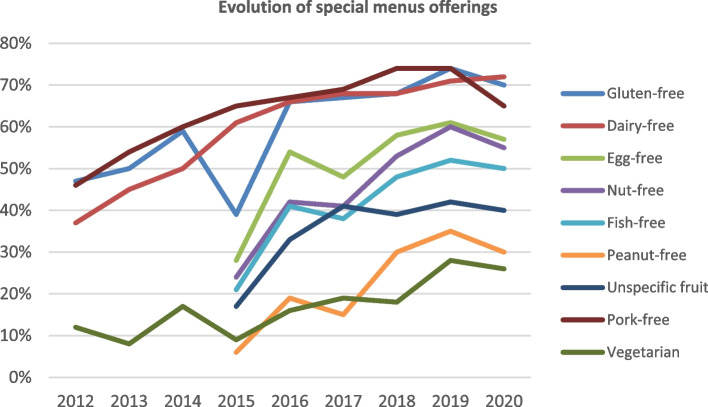
Evolution of the percentage of schools that offer special menus, by menu typology, in the School Menu Review Programme (PReME), 2012–2020

During the period 2012–2020, an increase was observed in the percentage of schools offering special menus in response to health requests or related to ethical or religious aspects (gluten-free, dairy-free, egg-free, nut-free, fish-free, peanut-free, without specific fresh fruits, pork-free or vegetarian) (Fig. 2). No statistically significant differences were detected by school category with respect to the overall availability of special menus catering to those with medical conditions (88.8% and 91.6% in state and private schools, respectively; *p* = 0.077; Table 4), the plans to avoid cross-contamination, or in providing details of special menus to families. However, private schools presented a greater range of menus for food allergies or intolerances and also allowed children to bring food prepared at home in a lunch box. In contrast, these schools reported a lower percentage of menus adapted for ethical or religious reasons (pork-free menu, vegetarian menu, etc.).

**Table 4:** Comparison by school category, state or private (private or subsidised schools), the percentage of schools that offer special menus for food allergies or intolerances, and menus adapted to special situations in the School Menu Review Programme (PReME), 2012–2020

	**State**	**Private**^**a**^	*P*^b^
	n (%)	n (%)	
***Special menus for medical conditions***			
Yes	1931 (88.8)	792 (91.6)	0.077
Celiac disease	1226 (54.3)	588 (66.7)	< 0.001
Diabetes	51 (2.3)	22 (2.5)	0.792
Crustaceans (allergy)	480 (21.2)	266 (30.2)	< 0.001
Molluscs (allergy)	381 (16.9)	230 (26.1)	< 0.001
Fish (allergy)	817 (36.2)	422 (47.8)	< 0.001
Eggs (allergy)	1014 (44.9)	492 (55.8)	< 0.001
Soy (allergy)	279 (12.3)	148 (16.8)	0.001
Milk (allergy)	1414 (62.6)	648 (73.5)	< 0.001
Peanuts (allergy)	453 (20.0)	262 (29.7)	< 0.001
Nuts (allergy)	925 (40.9)	427 (48.4)	< 0.001
Fresh fruit (allergy)	682 (30.2)	331 (37.5)	< 0.001
Other	50 (2.2)	24 (2.7)	0.476
***Cross-contamination plan***	129 (5.7)	44 (5.0)	0.478
***Adapted menus***			
No meat	893 (39.5)	251 (28.5)	< 0.001
No pork	1597 (70.7)	525 (59.5)	< 0.001
Vegetarian	423 (18.7)	156 (17.7)	0.533
Lactovegetarian	75 (3.3)	10 (1.1)	0.001
Vegan	37 (1.6)	8 (0.9)	0.167
Other	41 (1.8)	10 (1.1)	0.230
***Planning for families***	1768 (80.9)	661 (77.8)	0.016
***Lunch box option***	113 (5.1)	86 (9.9)	< 0.001

^a^ Private or subsidised schools

^b^ *P*-value associated with the χ^2^ statistic

**Fig. 3:**
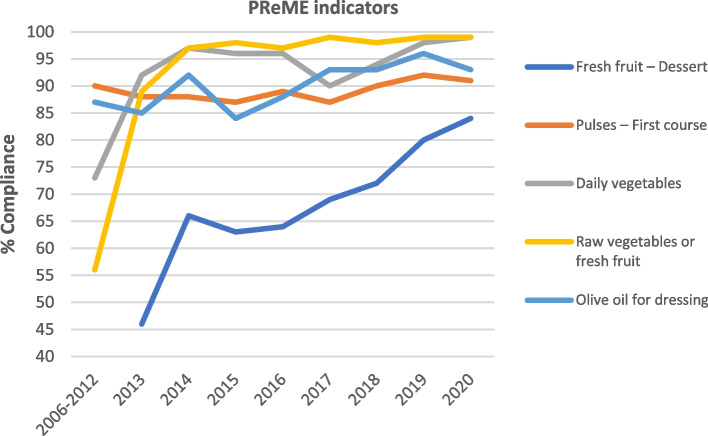
Evolution of the percentage of schools that comply with the recommendations for the key indicators of the School Menu Review Programme (PReME), 2006–2020

**Fig. 4:**
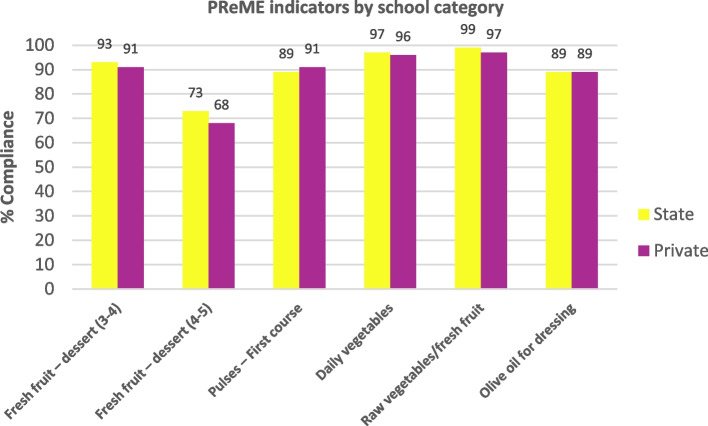
Comparison by school category – state or private (private or subsidised schools) – of compliance rates with the recommendations for indicators of the School Menu Review Programme (PReME), 2006–2020

Figure 3 shows the evolution of the percentage of schools that complied with dietary recommendations in relation to the five PReME indicators; this percentage steadily increased since the programme began in 2006, with a clear improvement in PReME’s initial years. The positive trend continued after 2012, with a very high compliance rate (over 70% in all the indicators and over 85% for 4 of the 5 indicators), and with statistically significant differences in the levels of compliance during this period, with the exception of pulses, which showed very high compliance rates for the entire study period (87–92%). For PReME indicators based on school category, although compliance was high in both cases, higher values were observed for state schools, with the exception of pulses (where compliance was slightly higher in private schools) and the use of olive oil as a dressing (with no significant differences) (Fig. 4).

**Fig. 5:**
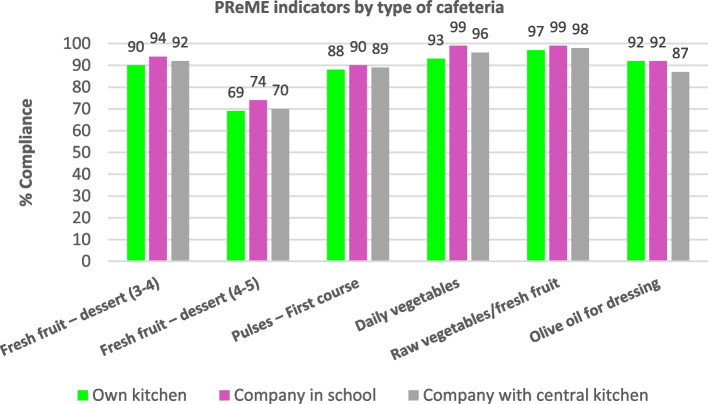
Comparison by type of cafeteria management (Own kitchen = schools with their own kitchen that prepares the meals; Company in school = company that cooks at the school; Company with central kitchen = company that supplies from a central kitchen) and compliance rates with recommendations using indicators of the School Menu Review Programme (PReME), 2006–2020

Differences are observed in the percentage of compliance with the recommendations based on the type of kitchen management. Thus, companies that cook at the school show higher compliance rates, with statistically significant differences, except for pulses and the presence of fresh foods on the menu (Fig. 5).

Regarding compliance with the recommended food frequencies, in general, and as shown in Table 5, an improvement was observed throughout the entire study period (2006–2020), with statistically significant differences for all items (*p* < 0.001), except for pulses whose compliance had been high since the beginning of the study (*p* = 0.216). A high level of compliance and maintenance over time was observed in the frequency of consumption for foods such as rice, pulses and vegetables as a first course. Although the level of compliance with pasta decreased notably after 2013, overall, the level of compliance increased during the study period. Compliance was high and rising for fish, total meat and fried foods, and moderately high and, above all, increasing for the recommended frequency of eggs after 2017. It is also interesting to note the increase in the compliance rate regarding the frequency of precooked foods and side salads in recent years. Compliance with the recommended frequency for fruit as a dessert started at 46% due to the change of recommendation in 2010 (from 3–4 to 4–5 times per week for fresh fruit), and increased steadily to the present level of 95%. In the case of red and processed meats, variable results were observed in terms of compliance with the recommended frequencies. An increasing trend in compliance was seen, although very high levels were not observed due to the short time elapsed since the issuing of the new recommendation in 2015.

**Table 5:** Evolution of the percentage of schools complying with the recommended frequencies for foods included in the School Menu Review Programme (PReME), 2006–2020

	**2006–2012**	**2013**	**2014**	**2015**	**2016**	**2017**	**2018**	**2019**	**2020**	*P*^a^
	(*N* = 1444)	(*N* = 240)	(*N* = 457)	(*N* = 440)	(*N* = 440)	(*N* = 350)	(*N* = 618)	(*N* = 487)	(*N* = 266)	
	n (%)	n (%)	n (%)	n (%)	n (%)	n (%)	n (%)	n (%)	n (%)	
**Food groups**										
*First courses*										
Rice	1192 (83.2)	208 (86.2)	404 (88.2)	377 (86.0)	371 (85.0)	304 (87.0)	543 (88.0)	444 (91.0)	246 (92.0)	< 0.001
Pasta	1330 (92.9)	86 (61.2)	162 (35.3)	143 (33.0)	168 (38.0)	169 (48.0)	345 (56.0)	300 (61.0)	179 (67.0)	< 0.001
Pulses	1289 (90.0)	211 (87.6)	411 (89.7)	379 (87.0)	389 (89.0)	303 (87.0)	553 (90.0)	446 (92.0)	242 (91.0)	0.216
Vegetables	1251 (87.3)	217 (90.1)	422 (92.1)	391 (90.0)	399 (91.0)	316 (90.0)	583 (94.0)	462 (95.0)	241 (91.0)	< 0.001
*Second courses*										
Fish	1223 (85.4)	196 (81.4)	442 (96.4)	415 (95.0)	417 (95.0)	329 (94.0)	596 (97.0)	480 (98.0)	261 (98.0)	< 0.001
Eggs	884 (61.7)	161 (66.9)	361 (78.9)	346 (79.0)	336 (76.0)	234 (67.0)	513 (83.0)	429 (88.0)	241 (91.0)	< 0.001
Total meat	1114 (77.8)	198 (82.1)	435 (94.9)	420 (96.0)	397 (90.0)	327 (93.0)	582 (94.0)	470 (96.0)	258 (97.0)	< 0.001
Red and processed meat					280 (64.0)	143 (41.0)	348 (56.0)	302 (62.0)	178 (67.0)	< 0.001
*Side dishes*										
Salad	668 (47.0)	116 (48.0)	273 (60.0)	264 (61.0)	258 (59.0)	177 (59.0)	395 (64.0)	324 (67.0)	162 (61.0)	< 0.001
*Desserts*										
Fresh fruit	1231 (86.0)^b^	112 (46.3)	287 (63.2)	273 (63.0)	280 (64.0)	242 (69.0)	441 (72.0)	390 (95.0)	223 (84.0)	< 0.001
*Cooking techniques*										
Precooked			339 (78.4)	60 (47.0)	70 (54.0)	61 (54.0)	111 (72.0)	94 (77.0)	48 (72.0)	< 0.001
Fried			423 (97.6)	405 (93.0)	412 (94.0)	341 (98.0)	592 (96.0)	462 (95.0)	253 (95.0)	< 0.001

^a^ *P*-value associated with the χ^2^ statistic

^b^ Fresh fruit 3–4 times a week

**Fig. 6:**
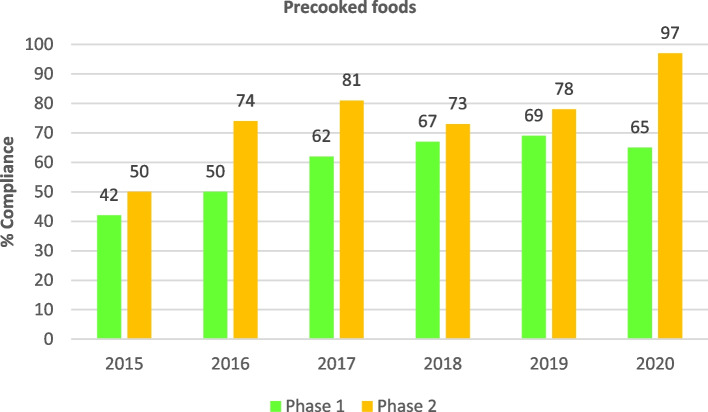
Evolution of the percentage of schools complying with recommendation to not include more than three precooked foods per month in meals between the initial review report (phase 1) and the follow-up report (phase 2) of the School Menu Review Programme (PReME), 2013–2020

**Fig. 7:**
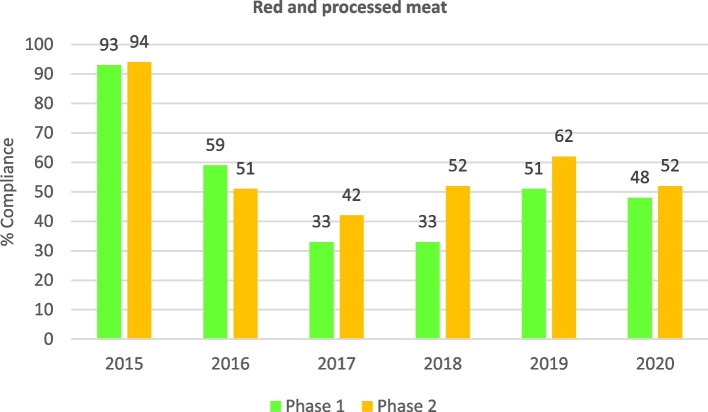
Evolution of the percentage of schools complying with recommendation to not include more than one portion of red or processed meat per week in meals between the initial review report (phase 1) and the follow-up report (phase 2) of the School Menu Review Programme (PReME), 2015–2020

With respect to the comparison of results obtained in PReME phase 1 (initial evaluation) reports and the phase 2 report (follow-up evaluation) for the period 2013–2020, an improvement can be observed in the level of compliance with the PReME recommendations in the follow-up report. This indicates that the PReME recommendations and suggestions for the improvement of meal plans reviewed after the initial report were being implemented, with statistically significant differences. Rising compliance rates were observed with respect to the recommendation to include uncooked vegetables or fresh fruit in the meals on a daily basis in both phases, reaching 99% in the last year. The percentage of schools that complied with the recommendation to include vegetables on the menu every day also showed an upward trend in both phases, reaching 100% in the last two years. The percentage of schools that complied with the recommendation to include no more than three precooked foods per month on the menu increased from 42 to 69% according to the initial report, while the follow-up report showed a maximum compliance of 97% in 2020 (Fig. 6). The percentage of schools that complied with the recommendation to include fresh fruit for dessert 4–5 times a week steadily increased during the period, obtaining a maximum compliance rate of 96% in the follow-up reports for 2020 versus 86% in the initial report. The percentage of schools that complied with the recommendation to include a maximum of one portion of red or processed meat per week in menus showed a significant decrease in 2016, coinciding with the application of new criterion by schools (Table 2); it was observed that the compliance rate was lower in follow-up reports than in the initial reports. On the other hand, in 2018, the compliance rate improved by almost 20 points between the initial and follow-up reports for this specific year (Fig. 7). The percentage of schools that complied with the recommendation to use olive oil or high oleic sunflower oil for cooking and frying showed increasing values, attaining compliance rates of 82% and 55%, respectively. In contrast, the percentage of schools complying with the recommendation to use olive oil for dressing remained very high throughout the entire period, reaching 99% in 2018 in the follow-up report. That same year, the initial reports showed a compliance rate of 96%. Improved compliance was observed in the follow-up report for all three types of oils utilized in school settings.

During the period of time that the PReME programme has been implemented, the major areas detected that required increased compliance were that olive oil should be more regularly used when cooking. In addition, although substantial improvements have been noted in recent years, compliance with the frequency of salads as a garnish and fruit as dessert could be further increased. It is also necessary to continue improvements regarding reduced frequency of pre-cooked dishes and, above all, reducing the presence of red and processed meats.

## Discussion

Healthy eating in childhood can promote optimal growth and development, better learning, and improved health and well-being. In general, childhood is a crucial period for establishing lifelong healthy eating habits [16, 33–37, 39]. As school breakfast and lunch can contribute to more than 50% of children’s calorie intake on school days [41, 44], by offering them healthy meals and nutrition education, schoolchildren can improve their diets, develop healthier eating habits and pass them on to their families and communities [39, 41, 47, 59–64]. A total of 39% of pre-school and primary school students use the school cafeteria service, according to data collected in 2017 in Spain [65]. In Catalonia, 42.5% of pupils use the school cafeteria service, which accounts for 10% of the total meals consumed during the year [66]. With the goal of improving the quality of the meals served in school cafeterias, PReME not only offers to review meal plans but also publishes and disseminates recommendations to facilitate the uptake of healthy eating habits.

In this study, we have analysed the data obtained from the assessment of school menus plans in Catalonia through the PReME programme from 2006 to 2020.

It is important to note that in the 14 years of PReME’s implementation, the review criteria have been updated on several occasions to align with new published scientific evidence, consensuses and recommendations (Table 2). In this respect, we highlight that reference documents in the field of child and school nutrition have been published, as well as new studies addressing the different types of food served in school cafeterias (red and processed meats, for example). As a result, in 2010, the recommendation was changed from fresh fruit for dessert 3–4 times a week to 4–5 times a week. Along the same lines, in 2015, following the WHO report on the carcinogenic potential of consuming red and processed meat [67], meat started to be classified as white meat or red and processed meat (until then, all these meat types were grouped under the generic term “meat”) and the recommended frequency of such meats was limited to a maximum of one portion per week. Likewise, the option of incorporating plant-based proteins (pulses and derivatives) for the second course was included. As a result of the priorities detected by the PReME team that were based on new diet trends or concerns, as well as the evolution of results observed over the years, preference has been given to including certain measurable aspects, such as assessing the utilization of precooked products, over other considerations. It is important to bear in mind that 30% of schoolchildren’s daily sodium intake is provided by the midday meal, and 9% of the sodium intake comes from precooked foods and processed meats included in school menus [68]. Studies carried out in the USA suggest that the meals and snacks served in schools include excessive amounts of saturated fat, sodium and sugar [51]. A number of studies based on the nutritional assessment of school meals in Spain have shown that the meals’ average sodium content is well above the recommendations in all cases [65, 69–71]. Therefore, in order to achieve the WHO target of a 30% reduction in mean salt intake by 2025, it is necessary to increase the consumption of unprocessed foods and reduce the presence of processed meats and precooked foods [72].

These changes in meal assessment criteria account for the fluctuations in compliance with certain recommendations over the years. For example, the notable decrease in compliance with the pasta recommendation in 2010 was due to the change in criteria linked to the publication of the DoCACE (Consensus Document on School Meals), which recommended serving pasta only once a week, albeit compliance has improved over the years. All of these new criteria were published on the PReME website and included in the 2017 version of the “Healthy eating at school” guide.

### Compliance with PReME indicators

The percentage of schools complying with the dietary recommendations encompassed in the five PReME indicators has followed an upward trend since the beginning of the programme. Compliance was seen to be higher in state schools with respect to the inclusion of fruit and cooked and uncooked vegetables as part of the daily menu. Regarding the presence of red and processed meats and olive oil for cooking and dressing, the results are given for the period comprising 2015–2020. This is due to the fact that changes were made in 2015 to the meal plan review criteria that affected these indicators (Table 2).

### Indicators based on the type of school category

In line with our evaluation, the study analysing compliance with NAOS indicators (DoCACE) in Guipuzcoa (fresh fruit ≥ 4 portions/week; vegetables ≥ 4 portions/week; pulses ≥ 1 portion/week; fish ≥ 1 portion/week; and precooked foods ≤ 1 portion/week), also showed that state schools attained the highest compliance rates for vegetables and precooked foods, while in the case of pulses, fish and fruit, compliance was identical for both state and private schools [73]. In a study assessing meals offered in school cafeterias comparing state, private and subsidised schools in Seville in 2014, and based on the recommendations of the PERSEO guide (Pilot School Reference Programme for Health and Exercise against Obesity**)** [74], it was observed that the state schools aligned more closely with the established recommendations. However, a higher proportion of state schools offered excessive quantities of meat products compared with subsidised or private schools, and the differences with respect to state schools were significant. With respect to the provision of pasta and rice, state and private schools delivered the recommended amount, while 35% of subsidised schools exceeded the recommendation. On the other hand, private schools showed closer alignment with recommendations addressing vegetables. This difference was significant with respect to the other two types of school. In addition, significant differences were observed in the three types of school with respect to pulses, with subsidised schools showing the closest alignment to recommendations [74]. In a study conducted in Asturias that assessed the availability of different food groups in school meals in the 2015–2016 academic year, a higher compliance rate was observed in state schools regarding the availability of fried foods, lower availability of fatty meats, greater availability of vegetables as a first course and a lower presence of animal protein in the first course [75].

### Indicators based on the type of cafeteria management

The type of cafeteria management had a significant influence on compliance with the indicators. Thus, companies that prepared meals onsite at schools showed statistically significant higher compliance rates, except for pulses and the presence of fruit and uncooked vegetables on the menu. Contrary to our results, in the studies carried out in Madrid [76] and Granada [71], no significant differences were found in the overall quality of meals prepared in school kitchens or by catering services. However, the latter offered more meat and less fish, eggs and fruit. In the Asturian study and in another study carried out in the region of Marina Alta in the Valencian Community, better results were obtained with meals prepared in school kitchens [75, 77]. In a later study by Llorens-Ivorra et al. in the Valencian Community, it was observed that the meals designed by the school itself contained more meat; and the meals prepared by catering companies contained more salad, vegetables, pulses, fish, oily fish, precooked foods and eggs. This difference could be due to the fact that the latter employ professional dieticians [27]. In contrast, a study conducted in 209 schools in 19 Spanish provinces in 2011, using criteria similar to those of the DoCACE, found that the quality of the meals prepared by catering services was superior to those prepared in-house, and improved when they were prepared jointly [78]. In the Asturian study, which assessed the availability of the different food groups in school meals during the 2015–2016 academic year, a higher level of compliance was observed for in-house preparation of precooked foods, pulses, greater availability of salads and vegetables as well as a lower availability of processed meat and animal protein in the first course. In addition, a higher level of compliance was observed for catering companies with respect to the availability of canned foods and fatty meat [75]. In the study by Arroyo et al. on the nutritional composition of school meals in Alicante, better quality was observed in the schools with their own kitchen [79]. On the other hand, in the nutritional comparison of the meals prepared in Granada, based on whether or not catering services were used, a higher calorie and carbohydrate content was seen in the schools with their own kitchen. However, no differences are observed with respect to other macronutrients [71].

### Compliance with recommended food frequencies

Compliance with the recommended frequencies of foods improved throughout the period, attaining statistical significance for all items except pulses, whose compliance had been high since the beginning of the study. Unlike our evaluation, an assessment conducted in schools in a municipality of Madrid in 2011 to assess the trend in the inclusion of vegetables in school meals (2004–2008) showed a decrease in the number of first courses with vegetables throughout the study period (26.2% in 2005; 11.9% in 2008) [80]. Likewise, in a 2014 study conducted in Madrid assessing compliance with the DoCACE recommendations, compliance rates lower than ours were observed for rice, pasta, fish, eggs, salad as a side dish and fruit as dessert, although the results were similar with respect to the availability of meat, which was above the recommended level [76]. Unlike our study, the availability of vegetables, fruit and pulses in school cafeterias in Seville and Asturias was insufficient. However, the findings were similar with respect to excessive amounts of meat [74, 81]. In the case of Asturias, the positive trend in most food items during the period 2011–2016 was attributed to the implementation of the “Healthy and organically produced food programme in school canteens”, based on the DoCACE [81]. In the study subsequently carried out in Asturias that assessed the availability of different food groups in school meals during the 2015–2016 school year, all schools obtained high compliance rates in all variables, except for the availability of precooked foods, vegetables as a first course, processed meat and the use of olive oil [75]. In a study conducted in the Valencian Community to evaluate dietary balance, it was observed that more than 60% of meals complied with the recommendations for salad, cooked vegetables, meat derivatives, precooked fish, eggs and sweet desserts, while less than 40% of meals complied with the recommendations for pulses, meat, oily fish, fried foods, dairy products and fruit [27]. However, this study used a questionnaire developed ad hoc as an instrument for evaluating the dietary balance of meals provided. This questionnaire did not coincide exactly with the NAOS indicators or with those of our study or the guides used in other autonomous regions of Spain. A study conducted in the Community of Castile and Leon, which used a questionnaire based on a regional guide and the DoCACE, non-compliance was observed with the recommendation regarding excessive intake of meat derivatives, dairy products and precooked food; non-compliance was also due to insufficient intake of fish, fruit, pulses and vegetables and salads as side dishes; there was also elevated use of fried, battered and breaded foods [82]. In the study of school cafeterias in the province of Vizcaya in the Basque Country, based on the DoCACE recommendations, it was observed that 100% of meals complied with the indicators relating to pulses, fish, meat, meat products and precooked dishes; however, non-compliance was observed with indicators relating to fresh fruit and vegetables [83]. Similarly, the study of school meals in the province of Granada concluded that the availability of the different food groups in the evaluated meals was largely in line with DoCACE guidelines, except for fruit [84]. For six canteens in the metropolitan area of Bilbao, analysed in 2010, it was found that the availability of vegetables, fish, eggs and fruit was below the recommendations and that of meat and dairy products exceeded recommendations [85].

However, school meals provided in the province of Guipuzcoa complied with the recommendations for meat portions and meat derivatives [73]. There are few studies that categorise processed meats or meat derivatives as a separate food group. In our study, an improvement in compliance was observed since red and processed meats were differentiated from white meats, with 67% of schools complying with the recommendation. Llorens-Ivorra et al. [27] observed a similar situation in the Valencian Community, with 70.2% of meals complying with the recommendation (≤ 3 portions of meat derivatives per week). In their study of school meals in Castile and Leon, De Mateo et al. observed a non-compliance rate of 97.2% (≤ 2 portions of meat derivatives per week) [82].

Most studies recommend an increase in the availability of fresh fruit, vegetables, pulses, fish and eggs, and a decrease in the provision of meat [27, 71, 73–76, 79–83, 85–87]. Globally, the average intake of fruit and vegetables is below the World Health Organisation’s recommendations in all WHO regions [88]. Interventions applying the social-ecological model acknowledge the importance of structural influences on children’s fruit and vegetable intake, for example, in the availability or accessibility of fruit and vegetables at home or in settings frequented by children, such as schools [38]. Combined with policies to limit availability of other less healthy foods, such interventions may be more effective in augmenting the consumption of recommended foods [89]. Despite strategies to increase fruit and vegetable consumption, fruit intake among Spanish children is still insufficient [31, 90–92]. Most school meals served in cafeterias show insufficient inclusion of fresh fruit, which are replaced by an excessive availability of dairy or sweet desserts [83, 86, 87, 93]. However, in our study, 95% of the schools complied with the recommendation of 4–5 portions of fruit per week. A similar finding was obtained in the city of Barcelona, where compliance was 82% [94]. On the other hand, only 37% of meals complied with the recommendation of fresh fruit offered as dessert in the study of the Valencian Community [27], 32.4% in the Community of Madrid [76], and in the Guipuzcoa study, compliance was nil [73].

It should be considered that part of the differences in results presented by the distinct studies could be due to the variety of methodological instruments used to assess the meals. Both Asturias and Bilbao used the PERSEO programme’s school cafeteria guide. In contrast, in the study of school meals in the city of Barcelona, which is based on PReME criteria, slightly lower compliance was observed for most food items compared with our study for the same year [94].

### Comparison between phase 1 and phase 2

The comparison of the results obtained in the phase 1 report (initial review) and the phase 2 report (follow-up review) showed an improvement in the level of compliance with the recommendations in the follow-up report. This would indicate that the recommendations and suggestions for improvement of the meal plans reviewed after the initial report were implemented successfully. Another study that assessed school meals from 2007 to 2010 demonstrated the effectiveness of the strategy of assessing school meals and sending the results to schools, complemented by a series of individual recommendations. As a result, the nutritional features of the meals provided at the schools have improved significantly [79].

### Special menus

In our study, an increase was observed in the percentage of schools that offered special menus in response to requests based on medical considerations or those related to ethical or religious aspects. Public or private ownership of schools did not have a significant influence on the overall availability of special menus for medical conditions, plans to avoid cross-contamination or the willingness to plan special menus for families. Private schools presented a greater range of options for food allergies or intolerances and they also made it possible to bring food prepared at home in a lunch box. However, they offered fewer menus adapted to ethical or religious considerations. On the other hand, the results of a study by Ramos evaluating school cafeterias in the city of Barcelona showed a higher percentage of schools that offered special menus for all menu typologies, compared to our results for 2019 [94]. One possible explanation could be that the sample in our study comes from centers in both urban and rural areas, while Ramos' study exclusively includes the city of Barcelona. Studies have shown the differences in eating patterns among populations living in rural versus urban areas [95–98], which would lead to the adaptation of school menus to these distinct patterns.

### Strengths and limitations

Our study presents certain limitations. As it applied a pre-post study design, causality cannot be established, and the results on compliance with the recommendations should be interpreted with caution. The results reported each year were derived from different schools which were not followed every year and as such, yearly outcomes are not strictly comparable. However, the results are useful for assessing which aspects of school meals need to be improved. As a result, it is possible to suggest potential interventions for increasing compliance. Neither serving sizes nor the real consumption of portions were assessed, and evaluations were based on the information given in theoretical monthly meal plans provided by the schools. Our study did not include information on the meals’ nutritional or organoleptic aspects but was limited to analysing the types of food provided. The assessment of existing dietary interventions, standards or policies in schools was also exempt in our evaluation. Furthermore, the changes made in the review criteria applied during the study period prevented assessing the evolution of certain items from the onset of the programme.

The discrepancies observed between this study’s results and those of other studies may be due to the fact that the same criteria were not used to assess the meals, as well as differences in recommendations, varying food item grouping or categorisation criteria or the exclusion of given items. The present study does not present data of a cohort of schools followed over a period of years, but rather “cross-sectional” information gathered serially over a period of years. However, given the low figures of percentages with repeated observations, it was hypothesized that the impact of such a violation of assumed independence would be residual in the statistical tests. Lastly, due to the pandemic situation in 2020, PReME was not able to fully maintain planned assessments for all schools involved in the programme.

Among the study’s strengths is the fact that it constitutes the first comprehensive review of school meals over a period of fifteen years in a large sample of schools in Catalonia. Although it is hard to longitudinally estimate the coverage of our data, at 2020, 2221 out of the 2332 Catalan schools had participated in the programme at some point.

## Conclusions

Our study shows an improvement in the nutritional content of school meals in the schools included in the PReME programme for the period 2006–2020. Compliance with PReME indicators is elevated and increasing, although variations can be observed depending on school category and cafeteria management. An improvement was also observed in the compliance with recommendations evidenced by follow-up reports, which would indicate that the recommendations and suggestions for improvement are being implemented successfully. Furthermore, an increase was observed in the percentage of schools offering special menus in response to health conditions or requests related to ethical or religious aspects, as well as the progressive removal of FBVMs. The upward trend in compliance with PReME’s recommendations is evidence of the programme’s effectiveness. Our study highlights the success of strategies for implementing and disseminating recommendations to improve the nutritional quality of school meals and to promote healthy eating. It is extremely important to plan and assess the quality of school meals and further research along these lines is needed to continue improving school meals, as well as incorporating legislative changes and making operational policy decisions.

## Data Availability

The datasets generated and analyzed during the current study are not publicly available due technical reasons but are available from the corresponding author on reasonable request.

## Abbreviations

PReME: School Menu Review Programme
NCDs: Diet-related non-communicable diseases
WHO: World Health Organization
NAOS: Strategy for Nutrition, Physical Activity and Obesity Prevention
PAAS: Promotion of health through physical activity and healthy eating
ASPCAT: Public Health Agency of Catalonia
DN: Dietitian-Nutritionists
PHS: Public health specialists
DoCACE: Consensus document on nutrition in schools
PERSEO: Pilot School Reference Programme for Health and Exercise against Obesity
